# Surface Electromyography-Based Evaluation of Neuromuscular Recruitment Under Different Resistance Conditions During Water-Resistance Rowing

**DOI:** 10.3390/s26144546

**Published:** 2026-07-17

**Authors:** Liwa Sha, Wen Hsin Chiu

**Affiliations:** 1 Department of Sports Training, Jilin Sport University, Changchun 130022, China; liwa-sha@m111.nthu.edu.tw; 2 Department of Education and Learning Technology, National Tsing Hua University, Hsinchu 300, Taiwan; 3 Department of Kinesiology, National Tsing Hua University, Hsinchu 300, Taiwan

**Keywords:** surface electromyography (sEMG), neuromuscular control, muscle activation, water-resistance rowing machine, resistance modulation, exercise evaluation

## Abstract

**Highlights:**

**What are the main findings?**

- Muscle activation remained below 40% MVIC across all resistance conditions, indicating that water-resistance rowing primarily provides low-to-moderate intensity neuromuscular loading.
- Neuromuscular recruitment increased substantially at the highest resistance level, whereas activation differences among the first three resistance levels were relatively limited.

**What are the implications of the main findings?**

- Quantifying resistance-dependent muscle activation may help practitioners identify appropriate resistance settings for individualized training and rehabilitation programs.
- Integrating sEMG sensing with rowing exercise enables an objective assessment of neuromuscular adaptation and supports the development of intelligent exercise-monitoring technologies.

**Abstract:**

Water-resistance rowing machines are widely used in fitness and rehabilitation; however, the neuromuscular effects of different resistance levels remain unclear. This study investigated resistance-dependent muscle activation during water-resistance rowing using surface electromyography (sEMG). Participants performed rowing exercise under four water-resistance conditions (MIN, Level 2, Level 3, and MAX) while maintaining a fixed stroke rate of 40 cycles/min. sEMG signals from the biceps brachii, triceps brachii, deltoid, latissimus dorsi, rectus femoris, and tibialis anterior were collected using a Delsys wireless system (1000 Hz) and normalized to maximal voluntary isometric contraction (MVIC). Results revealed significant interaction effects between resistance level and muscle group. The biceps brachii exhibited the greatest activation across all resistance conditions, whereas overall muscle activation remained below 40% MVIC. Activation differences were limited among the first three resistance levels but increased substantially at the highest resistance level. These findings support the use of sEMG for quantifying resistance-dependent neuromuscular adaptations and optimizing exercise prescription monitoring.

## 1. Introduction

In recent years, wearable sensing technologies and intelligent exercise monitoring systems have been increasingly applied in sports science, rehabilitation engineering, and neuromuscular performance evaluation. Exercise equipment integrated with biomechanical sensing systems can provide real-time physiological and movement-related information for exercise prescription, training optimization, and rehabilitation monitoring. Among the various indoor exercise modalities, rowing exercise has gained considerable attention because it simultaneously recruits upper-limb, trunk, and lower-limb muscle groups while providing low-impact cyclic movement patterns suitable for both athletes and rehabilitation populations.

Water-resistance rowing machines are designed to simulate real on-water rowing conditions through fluid-based resistance generated by paddles rotating inside a water tank. Compared with conventional air-resistance rowing ergometers, which generate resistance primarily through fan-induced aerodynamic drag, water-resistance systems produce smoother velocity-dependent nonlinear resistance and reduced acceleration–deceleration fluctuations during the rowing cycle. In contrast to magnetic-resistance ergometers that provide relatively constant and mechanically predetermined loading, fluid-based resistance continuously changes according to paddle velocity and water-flow dynamics. Moreover, unlike sliding ergometers that permit greater lower-extremity displacement and closely reproduce on-water rowing mechanics, water-resistance systems emphasize smooth force transmission and may alter muscle recruitment strategies by reducing abrupt mechanical loading and joint impact [1,2,3,4]. Previous studies have suggested that fluid-based and low-to-moderate resistance exercise modalities may provide safer and more tolerable loading conditions for older adults, rehabilitation populations, and individuals with limited muscular strength because of their smoother resistance characteristics and reduced joint loading [5,6,7]. However, it should be noted that these previous investigations did not specifically examine water-resistance rowing machines. Therefore, whether water-resistance rowing exercise provides comparable neuromuscular and functional benefits remains unclear and warrants further investigation. Furthermore, appropriately prescribed low-to-moderate resistance exercise can promote beneficial adaptations in muscle hypertrophy, cardiovascular fitness, and bone mineral density [8,9,10]. Therefore, understanding the neuromuscular demands associated with different resistance levels is important for optimizing exercise prescription and rehabilitation applications. To date, direct evidence regarding the suitability of water-resistance rowing machines for rehabilitation-oriented exercise prescription and older-adult populations remains limited. Consequently, quantifying the neuromuscular demands imposed by different resistance conditions may provide an important physiological basis for future exercise prescription and clinical applications.

Surface electromyography (sEMG) has been widely used to evaluate muscle activation characteristics during rowing exercise [11,12,13,14]. sEMG signals represent the summation of motor-unit action potentials and provide valuable information regarding muscle recruitment order, contraction intensity, intermuscular coordination, fatigue development, and neuromuscular control strategies [15]. Advances in wearable sensing technologies have further expanded the application of sEMG in sports performance monitoring and intelligent exercise evaluation systems [16]. Beyond quantifying muscle activation amplitude, sEMG has been recognized as a powerful tool for investigating neural control strategies and motor-unit recruitment behavior during human movement [17]. Moreover, muscle coordination and intermuscular recruitment patterns can be effectively studied using multi-channel sEMG measurements, particularly when several muscles contributing to a movement task are simultaneously monitored [18,19,20]. Consequently, wearable sEMG sensing provides an objective framework for assessing resistance-dependent neuromuscular adaptations during rowing exercise.

Previous investigations comparing different rowing ergometer designs have demonstrated that modifications in ergometer mechanics substantially influence neuromuscular recruitment patterns. For example, sliding ergometers permit greater lower-extremity displacement and generally require increased leg contribution during propulsion, whereas fixed ergometers are associated with greater activation of upper-body and trunk musculature [3,4]. These findings indicate that alterations in rowing-machine design can substantially modify intermuscular coordination and movement strategies during rowing exercise. However, these previous studies primarily investigated air-resistance rowing systems and did not examine fluid-based resistance mechanisms. Because water-resistance rowing machines employ a distinct nonlinear resistance profile generated by paddle–water interactions, their neuromuscular characteristics cannot be directly inferred from findings obtained using fixed or sliding air-resistance ergometers. Therefore, quantifying muscle recruitment patterns during water-resistance rowing remains necessary for understanding the specific neuromuscular demands imposed by this exercise modality.

Despite the increasing popularity of water-resistance rowing machines, their biomechanical and neuromuscular characteristics remain poorly understood. Most previous rowing studies have focused on air-resistance ergometers, sliding ergometers, or on-water rowing conditions, whereas evidence regarding fluid-based resistance systems remains scarce. Because water-resistance rowing combines nonlinear velocity-dependent loading, smooth resistance transitions, and reduced joint impact, its neuromuscular responses may differ substantially from those observed in conventional rowing ergometers. To our knowledge, no previous study has systematically quantified resistance-dependent muscle recruitment patterns across multiple resistance settings in a water-resistance rowing machine using synchronized multi-channel sEMG measurements under controlled stroke-rate conditions. Most previous studies evaluated rowing exercise using self-selected exertion intensity, prolonged exercise duration, or uncontrolled rowing velocity conditions [4,19]. Consequently, users and coaches often rely primarily on subjective perception to adjust resistance levels during training. Subjective exertion-based resistance regulation may reduce training effectiveness and introduce inconsistencies in exercise prescription [20]. Because resistance modulation may alter motor-unit recruitment requirements and mechanical loading demands, quantifying muscle activation characteristics across different resistance conditions may provide a physiological basis for exercise prescription and resistance progression strategies. Although sEMG has frequently been used to quantify activation amplitude during rowing exercise, relatively few studies have interpreted resistance-dependent adaptations from a neuromuscular-control and motor-unit recruitment perspective [3,13,16].

To date, few studies have systematically investigated the influence of different resistance levels in water-resistance rowing ergometers on muscle activation characteristics using wearable sEMG sensing systems. The novelty of the present study lies in combining controlled resistance modulation with synchronized multi-channel sEMG measurements to characterize resistance-dependent neuromuscular recruitment patterns in a fluid-based rowing system, which has rarely been investigated in previous rowing biomechanics literature. Because neuromuscular recruitment patterns are highly dependent on rowing-machine mechanics and resistance-generation characteristics, direct evaluation of muscle activation during water-resistance rowing may provide information that cannot be inferred from previous studies using other ergometer designs. Therefore, the purpose of this study was to investigate resistance-dependent neuromuscular recruitment patterns during water-resistance rowing exercise by examining four resistance settings (MIN, Level 2, Level 3, and MAX) under a standardized rowing cadence of 40 cycles/min. Surface electromyographic activity of six major muscles was quantified to determine whether progressive resistance modulation alters muscle recruitment characteristics. It was hypothesized that increasing resistance levels would progressively increase neuromuscular recruitment demands and produce distinct muscle activation patterns. The present investigation was intended to establish a physiological foundation for future exercise prescription studies rather than directly evaluate rehabilitation efficacy.

## 2. Materials and Methods

### 2.1. Subjects

Twelve healthy university students (age: 20.3 ± 1.2 years; height: 174.8 ± 5.0 cm; body mass: 71.0 ± 6.2 kg) with at least one year of rowing ergometer training experience voluntarily participated in this study. An a priori statistical power analysis was performed using G*Power software (Version 3.1, Heinrich-Heine-University Düsseldorf, Germany). Assuming a medium effect size (f = 0.40), an alpha level of 0.05, and a desired statistical power of 0.80 for repeated-measures analysis of variance, the estimated minimum sample size required was 10 participants. Therefore, the recruitment of 12 participants exceeded the minimum requirement and was considered sufficient to detect meaningful differences in neuromuscular activation across resistance conditions. Moreover, previous electromyographic investigations of rowing biomechanics have commonly recruited between 8 and 15 participants because repeated-measures experimental designs substantially reduce between-subject variability and increase statistical efficiency [3,4,19]. Participants reported no history of musculoskeletal injury or neurological disorders within the previous year. All participants were instructed to avoid vigorous physical activity for at least 48 h before testing. Prior to participation, all experimental procedures, potential risks, and participant rights were explained. Written informed consent was obtained from all participants before data collection. A certified sports injury prevention specialist was present throughout the experiments to manage potential exercise-related injuries. The study was conducted in accordance with the Declaration of Helsinki and was approved by the Institutional Research Ethics Review Committee of National Tsing Hua University in Taiwan (10712HT092, 18 March 2021).

### 2.2. Water-Resistance Rowing Ergometer

An Apollo water-resistance rowing machine (Apollo V, First Degree Fitness, Taoyuan, Taiwan) was used in this study. The rowing ergometer provided four resistance levels (MIN–MAX). The system utilized a dual-tank fluid resistance mechanism in which paddle blades rotated inside the water tank during the drive phase to generate velocity-dependent fluid resistance and simulate realistic on-water rowing sensations. The Apollo AR rowing machine utilizes a twin-tank variable fluid resistance (TT-VFR) mechanism in which resistance is regulated by altering the amount of water transferred between the inner and outer tanks through a mechanical valve system. The manufacturer defines four resistance settings, namely MIN, Level 2, Level 3, and MAX, corresponding to progressively greater water engagement and fluid drag during rowing exercise. The manufacturer does not provide absolute values for water volume, resistance force, or drag coefficients associated with each setting. Therefore, the present study adopted the manufacturer-defined resistance settings directly. To maximize reproducibility, all experimental trials were conducted using the same rowing machine, identical initial water conditions, and standardized manufacturer-defined resistance settings. Because the manufacturer does not provide absolute resistance-force values or water-volume specifications for each resistance setting, the present investigation was unable to quantify the exact mechanical loading associated with each condition. Future investigations should directly quantify the resistance forces, power output, and hydrodynamic loading characteristics associated with each manufacturer-defined resistance setting to facilitate inter-study comparisons and improve experimental reproducibility.

**Figure 1:**
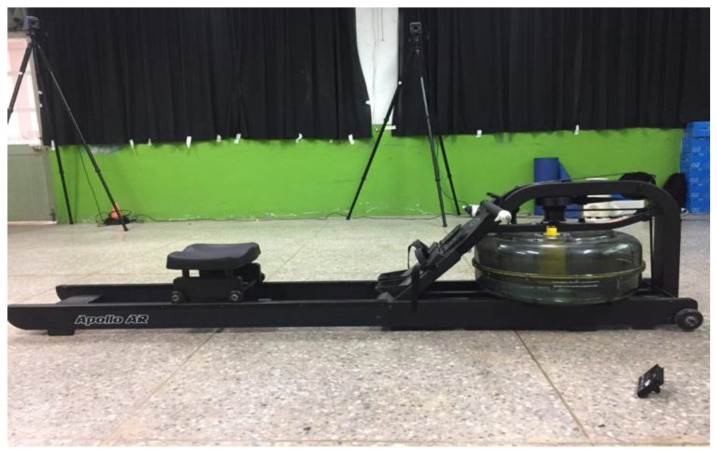
Water-resistance rowing ergometer employed for surface electromyography (sEMG)-based neuromuscular assessment. The Apollo V rowing ergometer (First Degree Fitness, Taiwan) generates fluid-based resistance through paddle–water interaction inside the water tank. Four resistance conditions were evaluated under a fixed stroke-rate protocol to quantify resistance-dependent muscle activation patterns during rowing exercise. Resistance was adjusted using the manufacturer-defined settings (MIN, Level 2, Level 3, and MAX), which progressively increased fluid engagement and rowing resistance.

**Figure 2:**
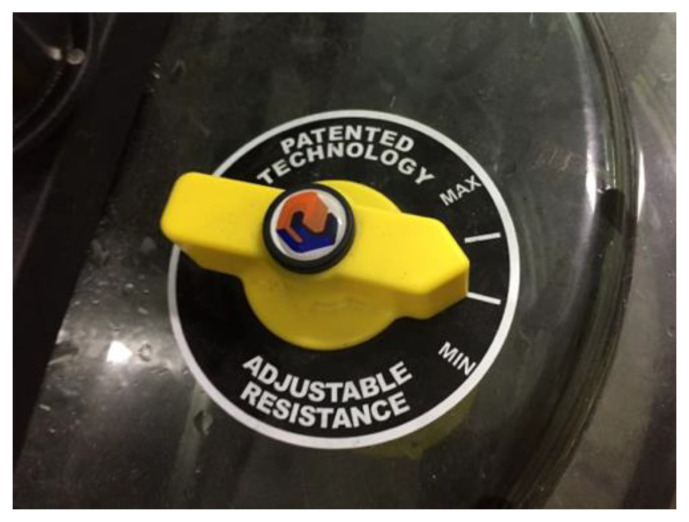
Apollo AR water-resistance rowing machine and resistance-level controller. The four manufacturer-defined resistance settings were MIN, Level 2, Level 3, and MAX.

Unlike conventional air-resistance rowing ergometers, the resistance generated by the water-resistance system depended on rowing stroke velocity and water flow dynamics. The Apollo AR water-resistance rowing machine provided four manufacturer-defined resistance settings, denoted as MIN, Level 2, Level 3, and MAX. For clarity and consistency, these manufacturer-defined labels are used throughout the manuscript. Specifically, the MIN and MAX settings correspond to the lowest and highest resistance conditions, respectively. Because the resistance controller of the Apollo AR rowing machine uses manufacturer-defined labels rather than numerical resistance values, the present study adopted these labels directly to avoid ambiguity and facilitate reproducibility. By systematically modifying fluid resistance while maintaining a constant stroke rate, the experimental design enabled a quantitative assessment of resistance-dependent muscle recruitment patterns during water-resistance rowing exercise. Resistance modulation was achieved by adjusting water volume between the inner and outer water tanks using an external control valve. The water-resistance rowing ergometer used in this study is illustrated in Figure 1, and the resistance controller is shown in Figure 2.

### 2.3. Rowing Cycle Definition

**Figure 3:**
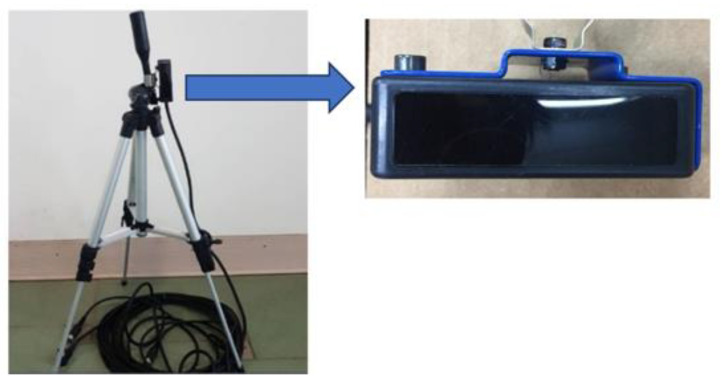
Infrared timing gate used for rowing-cycle segmentation. The infrared photoelectric sensor was mounted at the terminal position of the drive phase on the sagittal plane of the rowing ergometer. Beam interruption events were used to determine the transition between the drive and recovery phases, allowing for synchronized segmentation of rowing cycles and surface electromyography (sEMG) signals.

To standardize rowing movement patterns and minimize motion variability, the rowing cycle was divided into two phases according to previous rowing biomechanics studies [3]: the drive phase and the recovery phase. The drive phase began when the participant initiated lower-extremity extension and upper-extremity pulling actions and ended when the handle reached its posterior position. The recovery phase consisted of returning the handle and body to the starting position in preparation for the subsequent stroke. The present study focused exclusively on the drive phase for biomechanical and neuromuscular reasons. Previous rowing investigations have demonstrated that the drive phase represents the primary propulsive phase of the rowing cycle, during which the majority of mechanical work and force production are generated through coordinated activation of the lower extremities, trunk, and upper extremities [14,21,22]. Although muscle activity and postural control requirements are still present during the recovery phase, previous rowing biomechanics studies have demonstrated that the drive phase represents the primary propulsive period and accounts for the majority of external force generation and mechanical power production during rowing exercise [3,4,15]. Therefore, the present study focused specifically on the drive phase to investigate resistance-dependent neuromuscular recruitment under active loading conditions. The recovery phase was not ignored because of an absence of muscle activity, but rather because it represents a substantially lower external loading condition relative to the drive phase and was outside the primary scope of the present investigation. To facilitate automated rowing-phase detection, an infrared photoelectric timing gate was mounted at the end position of the drive phase (Figure 3). The sensor generated synchronized trigger signals whenever the participant interrupted the infrared beam during rowing movement. These trigger signals were integrated with the sEMG acquisition system and used to segment the rowing cycle into drive and recovery phases. This approach enabled precise temporal alignment between muscle activation signals and rowing kinematics, thereby improving the reliability of phase-specific neuromuscular evaluation.

**Figure 4:**
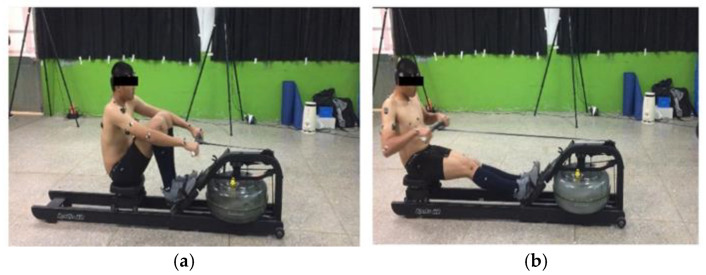
Rowing-cycle phase definition used in the present study. (**a**) Initial position of the drive phase, where the participant prepares to initiate the rowing stroke with flexed hips and knees; (**b**) terminal position of the drive phase, where the participant completes the pulling movement through lower-extremity extension, trunk extension, and upper-extremity flexion. The rowing cycle was divided into the drive phase and recovery phase for synchronized surface electromyography (sEMG) analysis.

Because resistance generation mainly occurred during the drive phase, only the drive phase was analyzed in this study. The drive phase was selected as the primary analysis window because it represents the force-producing period associated with resistance-dependent neuromuscular recruitment [23]. To standardize rowing movement patterns and minimize motion variability, the rowing cycle was divided into two biomechanically defined phases according to previous rowing biomechanics studies [1]: the drive phase and the recovery phase (Figure 2). The drive phase began when the participant initiated force production from the starting position by extending the lower extremities while simultaneously pulling the handle toward the trunk. During this phase, mechanical power was transferred from the lower extremities through the trunk to the upper extremities to overcome water resistance. The drive phase ended when the participant reached the terminal rowing position characterized by extended knees, a slightly reclined trunk, and the handle positioned near the lower thorax. Prior to testing, all participants received standardized rowing technique instruction to ensure consistent movement execution across all resistance conditions. The rowing cycle phases are illustrated in Figure 4.

### 2.4. Surface Electromyography (sEMG) Signal Acquisition and Processing

**Figure 5:**
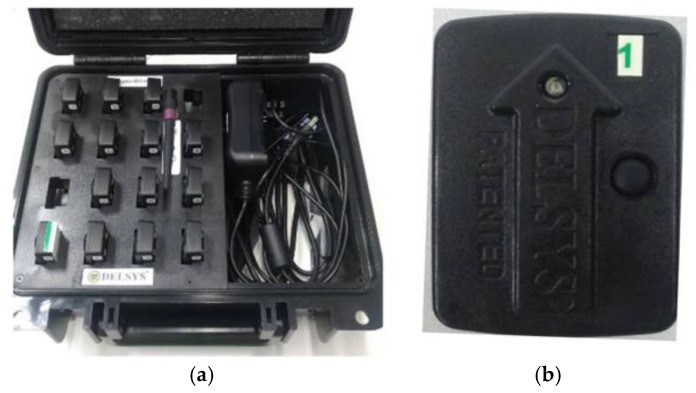
Wireless surface electromyography (sEMG) acquisition system used in the present study. (**a**) Delsys Trigno Wireless EMG System (Delsys Inc., Boston, MA, USA) including the base station, wireless sensors, and data acquisition components; (**b**) Trigno wireless sEMG sensor used for muscle activation measurements. The system sampled electromyographic signals at 1000 Hz and was synchronized with rowing-phase detection to quantify neuromuscular activation during rowing exercise.

Neuromuscular activity was recorded using a wireless surface electromyography (sEMG) system (Delsys Trigno Wireless EMG System, Delsys Inc., Boston, MA, USA). The system consisted of a base station, wireless sensors, charging modules, and data acquisition hardware for real-time physiological signal collection (Figure 5). Based on previous recommendations for sEMG signal acquisition and neuromuscular analysis, the sampling frequency exceeded the minimum requirement for avoiding aliasing effects during muscle activation recording [24]. Each Trigno wireless sensor incorporated differential surface electrodes and onboard signal amplification circuitry, enabling high-quality electromyographic signal acquisition while minimizing movement-related artifacts. Electromyographic signals were sampled at 1000 Hz and transmitted wirelessly to the acquisition workstation for subsequent processing and analysis. The wireless configuration reduced cable interference during rowing movements and allowed unrestricted execution of the rowing task. All acquired signals were synchronized with the infrared timing-gate system used for rowing-cycle segmentation, enabling phase-specific neuromuscular analysis during the drive phase of rowing exercise.

**Figure 6:**
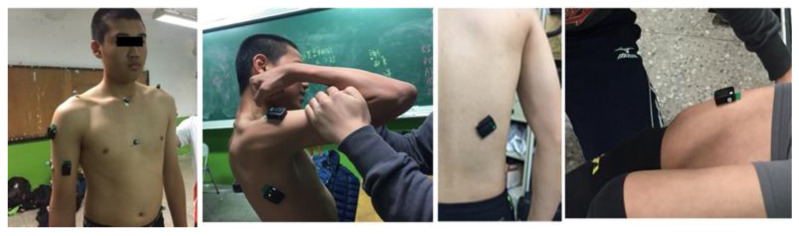
Placement of wireless surface electromyography (sEMG) sensors used for neuromuscular assessment during water-resistance rowing exercise. Wireless sEMG sensors (Delsys Trigno Wireless EMG System, Delsys Inc., Boston, MA, USA; sampling frequency = 1000 Hz) were attached to the dominant-side biceps brachii, triceps brachii, deltoid, latissimus dorsi, rectus femoris, and tibialis anterior muscles according to standardized electrode-placement guidelines. The recorded signals were synchronized with rowing-phase segmentation and subsequently used to quantify resistance-dependent muscle activation patterns during rowing exercise.

Surface electromyographic signals were recorded from six representative muscles located along the primary upper- and lower-extremity kinetic chains involved in rowing exercise, including the biceps brachii, triceps brachii, deltoid, latissimus dorsi, rectus femoris, and tibialis anterior (Figure 6). These muscles were selected because they represent major contributors to pulling propulsion, shoulder stabilization, and lower-extremity force transmission during rowing movements and have been commonly investigated in previous rowing electromyographic studies [3,4,15]. Additional muscles, including the erector spinae, hamstrings, and gastrocnemius, were not included because the primary purpose of the present study was to characterize representative resistance-dependent neuromuscular recruitment patterns rather than to perform a comprehensive whole-body muscle synergy analysis. Furthermore, limiting the number of sEMG electrodes reduced potential movement constraints and minimized motion artifacts associated with repetitive trunk flexion–extension and seat translation during rowing exercise. Before electrode placement, body hair and superficial skin keratinization were removed to reduce skin impedance and improve signal quality. Muscle activation amplitude was normalized to maximal voluntary isometric contraction (MVIC) according to previous electromyography normalization procedures [23,24]. The acquired sEMG signals were synchronized with the infrared timing-gate system used for rowing-phase segmentation. Following signal acquisition, the data were processed and analyzed to quantify resistance-dependent neuromuscular recruitment and muscle activation characteristics during the drive phase of rowing.

Following data acquisition, the sEMG signals were processed using a multi-stage signal-processing procedure. Raw signals were first band-pass filtered between 20 and 500 Hz to eliminate movement artifacts and unwanted frequency components. The filtered signals were then full-wave rectified and subsequently low-pass filtered at 10 Hz to obtain a smoothed linear envelope representing muscle activation profiles. Root mean square (RMS) calculations were performed to estimate the amplitude of muscle activation during rowing exercise. To facilitate inter-subject and inter-muscle comparisons, the RMS values were normalized to the corresponding maximal voluntary isometric contraction (MVIC) values and expressed as normalized muscle activation (%MVIC).

### 2.5. Experimental Control and Protocol

To improve measurement repeatability and reduce cycle-to-cycle variability, muscle activation data from the fourth to eighth rowing strokes were averaged for subsequent analysis. Furthermore, electrode placement followed standardized recommendations, muscle activation amplitudes were normalized to MVIC, and rowing cadence was controlled at 40 cycles/min throughout the experiment. Because the purpose of the present study was to evaluate resistance-dependent neuromuscular recruitment rather than fatigue development, the number of rowing strokes and recovery periods were intentionally designed to minimize acute fatigue accumulation. Previous rowing biomechanics studies demonstrated that rowing stroke rates between 34 and 40 strokes/min are commonly used in indoor rowing investigations [1,3,19]. Therefore, the rowing stroke rate was fixed at 40 strokes/min throughout all experimental conditions. Participants completed a 15 min dynamic warm-up before testing. An infrared synchronization device (Delsys Infrared Trigger System) was positioned 80 cm from the sagittal plane of the rowing ergometer to identify drive and recovery phases during rowing movement. To minimize psychological bias during resistance adjustment, the resistance control dial was visually covered using a black cloth. All participants practiced rowing under the fixed stroke-rate condition before formal testing until the investigators confirmed movement consistency and rhythm control.

A single-blind, counterbalanced design was employed throughout the experiment. To ensure standardized testing procedures, three investigators independently controlled the sEMG acquisition system, resistance-level adjustments of the water-resistance rowing ergometer, and metronome pacing, respectively. This approach minimized experimental bias and ensured consistent rowing performance across all resistance conditions. Participants performed rowing exercise under four resistance conditions (MIN–MAX). Each resistance condition consisted of only 11 rowing strokes, and participants rested for 3 min between resistance levels. However, only the fourth through eighth strokes were included in the subsequent analyses. The first three strokes were excluded because participants required several cycles to stabilize their movement rhythm, synchronize with the metronome, and achieve a consistent interaction with the fluid-resistance system. Similarly, the final three strokes were excluded to minimize potential influences associated with transient fatigue, reduced attention, and end-of-trial movement variability. Consequently, the fourth to eighth strokes were considered to represent the most stable steady-state rowing period and were therefore selected for analysis. The use of the middle five strokes reduced stroke-to-stroke variability and improved the reliability of resistance-dependent muscle activation measurements. To reduce signal variability and movement transition artifacts, only the 4th–8th drive-phase rowing strokes were included for further analysis. The experimental protocol was designed to minimize potential fatigue accumulation by limiting the number of rowing strokes and providing standardized recovery periods between conditions. However, no direct measurements of fatigue were obtained.

### 2.6. Statistical Analysis

An a priori power analysis was performed using G*Power software (Version 3.1, Heinrich-Heine-University Düsseldorf, Germany) before participant recruitment to determine the minimum sample size required for the repeated-measures experimental design. All subsequent statistical analyses were performed using SPSS Statistics 23.0 software (IBM Corp., Armonk, NY, USA). Descriptive statistics are presented as mean ± standard deviation. A two-way repeated-measures ANOVA was performed with the resistance level (MIN, Level 2, Level 3, and MAX) and muscle group (six muscles) as within-subject factors. Corresponding error terms and effect sizes (partial η^2^) were reported to facilitate the interpretation of statistical significance and practical relevance. When significant interaction effects were identified, simple main-effect analyses and Fisher’s protected least significant difference (LSD) post hoc comparisons were subsequently performed. Statistical significance was set at α = 0.05. In addition to *p*-values, partial eta squared (ηp^2^) values were calculated to evaluate the magnitude and practical significance of the observed effects. Effect sizes were interpreted as small (ηp^2^ = 0.01), medium (ηp^2^ = 0.06), and large (ηp^2^ ≥ 0.14) according to Cohen’s recommendations [23]. Post hoc pairwise comparisons were performed only when the corresponding simple main effects reached statistical significance. When significant interaction effects were observed, simple main effect analyses were performed, followed by Fisher’s protected least significant difference (LSD) post hoc comparisons. Statistical significance was set at α = 0.05.

## 3. Results

A two-way repeated-measures ANOVA demonstrated significant main effects of resistance level and muscle group as well as a significant resistance-by-muscle interaction (Table 1). Corresponding error terms and effect sizes are reported to facilitate evaluation of both statistical significance and practical relevance. Significant main effects were observed for resistance level (F = 8.314, *p* < 0.001, ηp^2^ = 0.430) and muscle group (F = 8.954, *p* < 0.001, ηp^2^ = 0.449). A significant resistance-by-muscle interaction was also identified (F = 2.703, *p* = 0.001, ηp^2^ = 0.197). According to Cohen’s criteria [23], the effects of muscle group and the interaction effect were large, whereas the effect of resistance level was medium-to-large. These findings indicate that resistance modulation produced meaningful changes in neuromuscular recruitment patterns and that the magnitude of these effects was not only statistically significant, but also practically relevant. A significant resistance-by-muscle interaction was observed, indicating that the influence of resistance modulation differed among muscle groups. Subsequent simple main-effect analyses revealed that only the biceps brachii demonstrated significant increases in activation across resistance conditions, whereas the remaining muscles exhibited relatively stable activation amplitudes despite progressive increases in resistance. These findings indicated that resistance modulation in the water-resistance rowing ergometer altered neuromuscular recruitment differently across muscle groups. Further simple main effect analyses revealed that only the biceps brachii demonstrated significant activation differences across resistance levels (F = 3.326, *p* = 0.020), whereas the triceps brachii (F = 0.352, *p* = 0.788), deltoid (F = 0.332, *p* = 0.802), latissimus dorsi (F = 0.298, *p* = 0.827), rectus femoris (F = 0.161, *p* = 0.923), and tibialis anterior (F = 0.292, *p* = 0.831) did not demonstrate significant inter-resistance differences. Within each resistance condition, significant differences among muscle groups were observed at Level 1 (F = 25.846, *p* < 0.001), Level 2 (F = 40.455, *p* < 0.001), Level 3 (F = 49.760, *p* < 0.001), and MAX (F = 65.111, *p* < 0.001). The statistical summary is presented in Table 1.

As shown in Table 2, the biceps brachii demonstrated progressively increasing activation amplitudes as the resistance level increased. The highest activation was observed at MAX (39.37 ± 22.00% MVIC), followed by Level 3 (34.81 ± 23.38% MVIC), Level 2 (30.61 ± 17.92% MVIC), and Level 1 (24.34 ± 13.35% MVIC). Subsequent simple main-effect analyses revealed that only the biceps brachii exhibited significant resistance-dependent increases in activation. The triceps brachii, deltoid, latissimus dorsi, rectus femoris, and tibialis anterior demonstrated relatively stable activation amplitudes across resistance conditions, and no significant pairwise differences were identified. The biceps brachii demonstrated significantly greater activation as resistance increased, with activation amplitudes progressively increasing from the MIN condition to the MAX condition. In contrast, although slight increases in mean activation amplitudes were observed in the triceps brachii, deltoid, latissimus dorsi, rectus femoris, and tibialis anterior, these differences did not reach statistical significance across resistance conditions. The deltoid demonstrated significantly greater activation at Levels 3 and 4 compared with Level 1, and Level 4 also demonstrated significantly greater activation than Level 2 (*p* < 0.05). The latissimus dorsi exhibited significantly lower activation at Level MIN compared with Levels 2 and MAX, while Level 4 demonstrated significantly greater activation than Level 3 (*p* < 0.05). For both the rectus femoris and tibialis anterior, MAX produced significantly greater activation amplitudes than the other resistance conditions (*p* < 0.05), suggesting increased lower-extremity neuromuscular contribution under high-resistance rowing conditions. Overall, activation increases were primarily observed under the highest resistance condition (MAX), whereas activation differences among MIN–3 were relatively limited.

Significant differences in muscle activation patterns were observed among muscle groups within each resistance condition. Across all resistance levels, the biceps brachii consistently exhibited the greatest activation amplitude and demonstrated significantly higher activation than the triceps brachii, deltoid, latissimus dorsi, rectus femoris, and tibialis anterior (*p* < 0.05). Overall, the neuromuscular recruitment pattern followed a relatively consistent hierarchy, with activation levels decreasing in the order of the biceps brachii, latissimus dorsi, tibialis anterior, rectus femoris, deltoid, and triceps brachii. Although the overall recruitment pattern remained similar across resistance conditions, minor variations were observed at higher resistance levels. Specifically, at Resistance Level 3, the rectus femoris exhibited the lowest activation amplitude among all examined muscles. At Resistance MAX, the deltoid demonstrated slightly greater activation than the rectus femoris, indicating a subtle shift in muscle recruitment strategy under increased resistance demands.

These findings indicate that water-resistance rowing predominantly recruits upper-extremity pulling musculature, particularly the biceps brachii and latissimus dorsi, during the drive phase of rowing. In contrast, the lower-extremity muscles and shoulder musculature demonstrated comparatively lower activation amplitudes under the fixed stroke-rate condition. This activation pattern suggests that the neuromuscular demands of water-resistance rowing are primarily concentrated on the upper-body pulling muscles responsible for force generation and resistance overcoming.

The resistance-modulated rowing conditions demonstrated distinct neuromuscular recruitment adaptations. At lower resistance conditions (Levels MIN–2), muscle activation amplitudes remained relatively low across all muscles, suggesting that rowing exercise was performed under low-to-moderate neuromuscular loading. As resistance increased, particularly at MAX, both upper- and lower-extremity muscles demonstrated elevated activation amplitudes. This finding suggests that increased water resistance required greater motor-unit recruitment to maintain the prescribed stroke frequency. Because normalized muscle activation remained below approximately 40% MVIC under all resistance conditions, water-resistance rowing exercise may represent a relatively low-to-moderate neuromuscular loading modality in healthy young adults. These findings suggest that water-resistance rowing may have potential applications in rehabilitation-oriented exercise prescription and low-intensity resistance-training programs; however, such applications remain speculative and should be confirmed in future studies involving clinical and older-adult populations.

## 4. Discussion

Because muscle activation analyses were restricted to the steady-state portion of each trial, the present findings primarily reflect stable resistance-dependent neuromuscular responses rather than transient adaptations occurring immediately after resistance transitions or near the termination of exercise. Because only the middle portion of each rowing trial was analyzed, the present investigation did not examine potential neuromuscular adaptations occurring during exercise initiation or termination. It should be acknowledged that the recovery phase may still involve low-to-moderate muscle activation associated with postural stabilization, segmental coordination, and movement deceleration. Therefore, the present findings should be interpreted specifically as resistance-dependent neuromuscular responses during the active propulsion phase of rowing exercise rather than as representations of the entire rowing cycle. Because only the drive phase was analyzed, potential neuromuscular contributions associated with recovery-phase stabilization and interphase coordination could not be evaluated. Future investigations should examine muscle activation patterns throughout the entire rowing cycle to obtain a more comprehensive understanding of rowing neuromuscular control. Future studies should also simultaneously quantify both drive- and recovery-phase muscle activation patterns to investigate phase-specific neuromuscular strategies and interphase coordination mechanisms during water-resistance rowing exercise.

The primary finding of the present study was that resistance modulation in the water-resistance rowing machine altered neuromuscular recruitment patterns during rowing exercise. Specifically, the greatest activation differences were observed between MIN and MAX resistance conditions, whereas the intermediate resistance levels demonstrated relatively limited activation changes across most muscles. This phenomenon may be associated with the fluid-resistance characteristics of the water-resistance rowing ergometer. Unlike conventional pin-loaded resistance machines, where resistance increments are mechanically discrete and relatively large, the water-resistance system modifies drag force by adjusting water volume and paddle–water contact area, thereby producing smoother and more progressive resistance changes. Consequently, small resistance adjustments may not sufficiently increase neuromuscular demand to induce substantial additional motor-unit recruitment. From a neuromuscular-control perspective, the observed changes in muscle activation amplitudes likely reflect alterations in motor-unit recruitment strategies rather than merely an increase in external force production. Surface electromyographic signals represent the summation of motor-unit action potentials and have been widely used to infer neural drive and recruitment behavior during dynamic movements [3]. Farina et al. [13] further suggested that multi-channel sEMG measurements provide valuable information regarding neural control strategies and motor-unit behavior during complex motor tasks. In the present study, the relatively limited activation differences among the first three resistance conditions suggest that similar neural recruitment strategies were maintained despite progressive increases in external resistance. In contrast, the substantial activation increase observed at the maximum resistance condition may indicate additional recruitment of motor units and enhanced neural drive to preserve movement performance under increased loading demands.

Previous studies have demonstrated that fluid-based resistance exercise systems provide smoother resistance profiles and lower joint-loading characteristics, making them particularly suitable for older adults, rehabilitation populations, and individuals with reduced muscular capacity [5,6]. From a neuromuscular perspective, the observed activation changes may reflect alterations in motor-unit recruitment strategies rather than merely increases in muscle force production. Surface electromyography has been widely recognized as an indirect measure of motor-unit recruitment and neural drive during human movement [15,24]. De Luca [24] proposed that surface electromyographic signals represent the summation of motor-unit action potentials generated during muscle contraction and therefore provide valuable information regarding muscle recruitment behavior and neuromuscular activation strategies. Furthermore, sEMG amplitude has been widely used as an indirect indicator of motor-unit recruitment and neural drive during dynamic movement tasks [15,24]. The relatively small differences in muscle activation observed among the MIN, Level 2, and Level 3 conditions may suggest that neuromuscular recruitment patterns remained relatively stable across these resistance settings. One possible explanation is that similar neural recruitment strategies were adopted under these lower resistance conditions. However, an alternative interpretation cannot be excluded. Because the present study did not directly quantify resistance force, power output, or motor-unit behavior, it is also possible that the resistance increments among the first three settings were insufficient to produce measurable physiological adaptations. Likewise, the substantially greater activation observed at the MAX condition may reflect increased neuromuscular demands and potentially greater motor-unit recruitment requirements. Nevertheless, the present data do not permit direct confirmation of the underlying neural mechanisms. Therefore, these interpretations should be regarded as plausible physiological explanations rather than definitive evidence of motor-unit recruitment strategies. Likewise, the substantially greater activation observed at the MAX condition may reflect increased neuromuscular demands and potentially greater motor-unit recruitment requirements. Nevertheless, the present data do not permit direct confirmation of the underlying neural mechanisms. Therefore, these interpretations should be regarded as plausible physiological explanations rather than definitive evidence of motor-unit recruitment strategies.

The present findings further support the potential application of water-resistance rowing as a progressive low-to-moderate intensity resistance training modality. Previous evidence has demonstrated that progressive resistance training improves muscle strength, functional capacity, and neuromuscular performance [25]. Progressive resistance exercise has also been shown to improve muscle function, physical performance, and functional recovery in individuals with sarcopenia, age-related muscle weakness, and post-surgical conditions [7,26,27]. Because muscle activation amplitudes under all resistance conditions remained generally below 40% MVIC, water-resistance rowing exercise may be classified as a low-to-moderate intensity neuromuscular training modality. Although muscle activation remained below 40% MVIC under all resistance conditions, this level of neuromuscular demand should not be interpreted as physiologically insignificant. Surface electromyography amplitudes below approximately 40% MVIC are generally considered indicative of low-to-moderate neuromuscular loading and are frequently associated with endurance-oriented motor-unit recruitment and submaximal force production [3,13]. Under such conditions, motor-unit recruitment is typically achieved primarily through activation of low-threshold motor units, resulting in lower metabolic stress and reduced mechanical loading on joints and connective tissues [13]. Previous studies have demonstrated that low-to-moderate resistance exercise can still induce meaningful physiological adaptations, including improvements in muscular endurance, functional performance, neuromuscular coordination, and exercise tolerance when appropriately prescribed [6,18,27]. Furthermore, progressive resistance interventions using relatively low mechanical loads have been shown to improve muscle function and physical performance in older adults and rehabilitation populations [7,17,28]. Although these findings suggest potential utility for rehabilitation-oriented exercise applications, the present study did not directly investigate older adults or clinical populations. Therefore, extrapolation of the present findings to rehabilitation settings should be interpreted cautiously and requires direct verification in future studies.

Although the exercise duration employed in the present study was relatively short, fatigue-related neuromuscular adaptations should still be considered when interpreting the findings. Previous studies have demonstrated that both central and peripheral fatigue mechanisms can alter motor-unit recruitment behavior, muscle coordination strategies, and neural drive during cyclic exercise [2,12]. Neuromuscular fatigue may induce the compensatory recruitment of additional motor units and modify intermuscular coordination patterns in order to maintain movement performance [12]. However, several experimental procedures were implemented to minimize fatigue-related influences in the present study. Each resistance condition involved only a limited number of rowing strokes, and a 3-minute recovery period was provided between conditions. Furthermore, muscle activation amplitudes remained below 40% MVIC across all resistance conditions, suggesting relatively low metabolic and neuromuscular loading demands. Therefore, the observed differences in muscle activation were likely attributable primarily to resistance modulation rather than fatigue accumulation. Although fatigue-related adaptations were not directly examined in the present investigation, fatigue cannot be completely excluded as a potential contributing factor. Nevertheless, the relatively short exercise duration and the low-to-moderate muscle activation amplitudes observed in this study suggest that substantial fatigue accumulation was unlikely. Because fatigue-related variables were not directly measured, the present findings should be interpreted as resistance-dependent neuromuscular responses rather than fatigue-induced adaptations. Although substantial fatigue accumulation was considered unlikely under the present experimental conditions, the absence of direct fatigue measurements precludes definitive conclusions regarding the contribution of fatigue to the observed muscle activation patterns.

Although numerical increases in activation amplitudes were observed in several muscles, these differences did not reach statistical significance. Consequently, resistance modulation appears to preferentially affect the biceps brachii rather than producing generalized increases in activation across all examined muscle groups. Another important finding of the present study was that the biceps brachii and latissimus dorsi consistently demonstrated significantly greater activation amplitudes than the tibialis anterior, rectus femoris, deltoid, and triceps brachii across all resistance conditions. The predominance of biceps brachii and latissimus dorsi activation may also indicate a coordinated neuromuscular strategy adopted during water-resistance rowing. Hug [16] proposed that muscle activation patterns should be interpreted as coordinated recruitment among multiple muscles rather than isolated activation amplitudes. During rowing exercise, propulsion is generated through sequential force transmission from the lower extremities to the trunk and ultimately to the upper extremities. Therefore, the elevated activation observed in the biceps brachii and latissimus dorsi may indicate that these muscles function as principal contributors to propulsion and resistance overcoming. The present findings suggest that water-resistance rowing is regulated through coordinated intermuscular recruitment strategies that preferentially emphasize upper-extremity pulling musculature under fixed stroke-rate conditions. The present findings should not be interpreted as indicating that all examined muscles responded uniformly to resistance modulation. Rather, the significant interaction effect suggests that resistance-dependent neuromuscular adaptations were muscle specific.

Although the triceps brachii demonstrated numerically greater activation at higher resistance settings, these increases were not statistically significant. Therefore, the present findings suggest that resistance modulation primarily influenced the biceps brachii, whereas the remaining muscles maintained relatively stable activation patterns under the present experimental conditions. Under fixed stroke-rate conditions, increases in resistance may primarily require greater upper-limb pulling force generation rather than proportional increases in the activation of all muscle groups. Consequently, additional neuromuscular demands may have been preferentially accommodated through increased recruitment of the biceps brachii, which serves as one of the principal contributors to handle pulling and force transmission during the drive phase. Similar activation tendencies have been reported in previous rowing electromyographic studies [3,4,19,29], which also identified substantial contributions of upper-body pulling musculature during rowing movements. However, direct comparisons should be interpreted cautiously because important methodological differences exist between studies. In particular, several previous investigations employed self-selected or uncontrolled stroke-rate conditions, whereas the present study standardized rowing cadence at 40 cycles/min. Because stroke rate has been shown to influence rowing mechanics, force production, and muscle activation patterns, the present findings should be regarded as qualitatively comparable rather than quantitatively equivalent to previous reports. Nevertheless, the observation that upper-limb pulling musculature demonstrated relatively greater activation across studies may suggest that this recruitment tendency is robust despite methodological variations in rowing protocols. The use of a fixed stroke rate in the present investigation was intentionally adopted to reduce movement variability and isolate the effects of resistance modulation on neuromuscular recruitment. Consequently, differences between the present findings and previous studies may partly reflect experimental design rather than physiological disagreement.

From a muscle-coordination perspective, the dominance of biceps brachii and latissimus dorsi activation may also reflect a specific intermuscular coordination strategy adopted during water-resistance rowing. Hug [18] suggested that muscle coordination can be effectively investigated using multi-channel sEMG measurements because coordinated activation among multiple muscles contributes substantially to movement efficiency and force transmission. During rowing exercise, force generated by the lower extremities is transferred through the trunk and ultimately expressed through upper-extremity pulling actions. The relatively low activation amplitudes observed in the tibialis anterior and rectus femoris may indicate that lower-extremity neuromuscular demands were comparatively modest under the present experimental conditions. One possible explanation is related to the mechanical characteristics of the water-resistance rowing machine. The seat-rail system may facilitate relatively smooth seat translation and potentially reduce the active lower-extremity demands required during rowing exercise. However, this interpretation should be considered speculative because seat displacement, seat velocity, lower-extremity kinematics, and mechanical loading variables were not directly quantified in the present investigation. Therefore, the observed activation patterns cannot be definitively attributed to the seat-rail mechanical design. Alternative explanations, including the relatively low resistance increments, individual rowing strategies, and participant experience levels, may also have contributed to the low activation amplitudes observed in these muscles. Future studies incorporating motion capture systems, inertial sensors, and seat-translation measurements are necessary to determine whether rowing-machine mechanics directly influence lower-extremity muscle recruitment patterns. The present findings support previous evidence suggesting that rowing-machine design substantially influences neuromuscular recruitment characteristics [3,4]. The upper-extremity dominant recruitment pattern observed in the present study may partly reflect the unique mechanical properties of fluid-based resistance and further suggests that neuromuscular responses obtained from air-resistance or sliding ergometers should not be directly generalized to water-resistance rowing systems. The observed medium-to-large effect sizes further suggest that resistance modulation exerted meaningful influences on neuromuscular recruitment and that these adaptations may have practical implications for exercise prescription and rehabilitation-oriented resistance progression.

Although sEMG provides valuable information regarding neuromuscular activation, correct interpretation of muscle activation patterns should be considered in conjunction with biomechanical characteristics of the movement task [30]. Disselhorst-Klug and Williams [30] emphasized that electromyographic signals alone cannot fully explain movement mechanics without the consideration of kinematic and kinetic variables. Therefore, the lower activation levels observed in the rectus femoris and tibialis anterior should not be interpreted as indicating limited functional involvement. Instead, these findings likely reflect the specific mechanical characteristics of the water-resistance rowing machine, including seat translation dynamics and fluid-resistance generation mechanisms. The relatively short exercise duration (11 strokes per condition) and the provision of a 3-minute recovery period between resistance conditions were intentionally implemented to reduce the likelihood of substantial fatigue accumulation. Furthermore, normalized muscle activation remained below approximately 40% MVIC under all resistance conditions, suggesting relatively modest neuromuscular loading demands. However, it should be acknowledged that fatigue-related variables, including perceived exertion, physiological fatigue markers, and electromyographic indicators of fatigue, were not directly quantified in the present investigation. Therefore, the possibility that minor fatigue-related adaptations occurred cannot be completely excluded. Accordingly, the assumption that fatigue effects were minimized should be interpreted cautiously and regarded as a reasonable experimental assumption rather than a directly verified finding. Because no subjective or physiological indicators of fatigue were collected, the present investigation cannot directly verify that fatigue accumulation was negligible. Consequently, minor fatigue-related influences on muscle activation patterns cannot be entirely excluded. The relatively stable activation amplitudes observed in the triceps brachii, deltoid, latissimus dorsi, rectus femoris, and tibialis anterior should not necessarily be interpreted as evidence of an absence of neuromuscular adaptation. Rather, these muscles may have fulfilled stabilizing, coordinative, or force-transmission functions that did not require substantial increases in activation magnitude under the present loading conditions. Future studies should investigate the interaction between resistance modulation and fatigue development during prolonged rowing exercise.

In particular, combining sEMG with biomechanical sensing technologies may further elucidate fatigue-related changes in motor-unit recruitment, intermuscular coordination, and movement-control strategies during water-resistance rowing. Although several muscles that contribute to rowing performance, including the erector spinae, hamstrings, and gastrocnemius, were not examined in the present investigation, the selected muscles adequately represented the principal upper- and lower-extremity kinetic chains associated with rowing propulsion. Nevertheless, additional muscle recordings may provide further insights into whole-body intermuscular coordination and muscle synergy strategies during water-resistance rowing exercise. Because only six representative muscles were examined, the present findings do not fully characterize whole-body neuromuscular coordination during rowing exercise. Future investigations incorporating additional trunk and lower-extremity muscles may provide a more comprehensive understanding of muscle synergy and movement-control mechanisms.

Although the recovery phase was not analyzed in the present study because of its relatively low external loading demand, future investigations should examine phase-specific muscle activation and intermuscular coordination throughout the entire rowing cycle to further elucidate neuromuscular control strategies during water-resistance rowing exercise. Because the present study primarily quantified muscle activation magnitude, future investigations should further examine motor-unit recruitment strategies, intermuscular coordination, and neural control mechanisms during water-resistance rowing exercise. Integrating sEMG measurements with biomechanical sensing technologies, including inertial measurement units and motion-capture systems, may facilitate a more comprehensive understanding of neuromuscular control adaptations under different resistance conditions. Future investigations should also examine the influence of different rowing velocities and stroke frequencies on neuromuscular recruitment patterns, investigate phase-specific muscle activation profiles and co-contraction behavior throughout the rowing cycle, and integrate sEMG with inertial measurement units, motion-capture systems, and other biomechanical sensing technologies. Such multidisciplinary approaches could provide a more comprehensive understanding of resistance-dependent movement control and facilitate the development of intelligent exercise-monitoring systems and evidence-based exercise prescription strategies.

The present findings further support the concept that resistance-dependent adaptations in rowing exercise should be interpreted from both biomechanical and neuromuscular perspectives. As proposed by De Luca [24], muscle activation patterns obtained from sEMG provide valuable information regarding motor-unit recruitment behavior, whereas Bento and Rodacki [31] demonstrated that low-impact water-based exercise can induce meaningful improvements in muscle function. Collectively, these findings suggest that water-resistance rowing may represent an effective training modality capable of simultaneously providing progressive neuromuscular stimulation and maintaining relatively low mechanical loading, thereby supporting both exercise performance enhancement and rehabilitation-oriented applications. Several limitations should be acknowledged. First, although experimental procedures were specifically designed to minimize fatigue accumulation, fatigue-related neuromuscular adaptations were not directly quantified using physiological or perceptual measurements. Therefore, the potential influence of fatigue on muscle recruitment patterns cannot be completely excluded.

The relatively limited activation differences among the first three resistance levels suggest that users may safely adjust resistance within these ranges without inducing substantial increases in neuromuscular demand. Consequently, these resistance conditions may be suitable during the early stages of rehabilitation or conditioning programs where gradual progression and movement familiarization are desired. In contrast, the highest resistance condition elicited substantially greater neuromuscular recruitment and may therefore represent a transitional stage toward higher-intensity resistance training and functional performance enhancement. The present findings may also have implications for intelligent exercise monitoring systems. Because resistance-dependent muscle activation can be objectively quantified using wearable sEMG technology, future rowing systems may integrate neuromuscular sensing and resistance-control algorithms to provide individualized exercise prescription, adaptive resistance progression, and real-time rehabilitation monitoring. Although the present findings suggest that water-resistance rowing may impose relatively low neuromuscular demands, direct recommendations for older adults and rehabilitation populations cannot be made based on the current data. Future investigations involving clinical populations are necessary to determine the safety, feasibility, and efficacy of water-resistance rowing exercise in these groups.

Future investigations should further examine the effects of different rowing velocities, stroke frequencies, and prolonged exercise durations on neuromuscular recruitment patterns during water-resistance rowing. More importantly, future studies should integrate surface electromyography with additional biomechanical sensing technologies, including inertial measurement units (IMUs), motion-capture systems, force sensors, and pressure-sensing platforms. Such multimodal sensing approaches may simultaneously quantify muscle activation, segmental kinematics, force transmission characteristics, and movement coordination strategies throughout the rowing cycle. The integration of wearable and biomechanical sensing technologies may facilitate a more comprehensive understanding of resistance-dependent neuromuscular adaptations and enable the development of intelligent rowing systems capable of providing individualized exercise prescription, adaptive resistance progression, and real-time rehabilitation monitoring. Such approaches may ultimately improve both sports performance evaluation and rehabilitation-oriented exercise applications.

Several limitations of the present study should be acknowledged. First, the sample size was relatively small and consisted exclusively of healthy young adults with previous rowing-machine experience. Therefore, caution should be exercised when generalizing the present findings to older adults, rehabilitation populations, elite rowers, or individuals without rowing experience. Second, although the experimental procedures were specifically designed to minimize fatigue accumulation by controlling the number of rowing strokes and providing sufficient recovery periods, fatigue-related neuromuscular adaptations were not directly quantified using physiological or perceptual measurements. Consequently, the potential influence of fatigue on muscle recruitment behavior cannot be completely excluded. Third, the present study focused exclusively on the drive phase because this phase represents the primary propulsive period and the principal resistance-loading component of rowing exercise. However, neuromuscular coordination during the recovery phase may also provide valuable information regarding movement control and rowing efficiency. Finally, only surface electromyographic signals were analyzed in the present investigation. Additional biomechanical information, including kinematic variables, joint kinetics, and inertial sensor measurements, was not collected. Future investigations integrating sEMG with motion capture systems, inertial measurement units, and kinetic analyses may provide a more comprehensive understanding of neuromuscular control strategies during water-resistance rowing exercise. Another limitation of the present study is that only sEMG measurements were collected. Additional biomechanical variables, including joint kinematics, segmental accelerations, force production characteristics, and plantar pressure distribution, were not simultaneously measured. Consequently, the underlying biomechanical mechanisms associated with resistance-dependent muscle recruitment could not be fully elucidated. Accordingly, the potential rehabilitation implications proposed in this study should be regarded as speculative and hypothesis-generating rather than clinically validated findings.

## 5. Conclusions

The present study investigated the effects of resistance modulation in a water-resistance rowing machine on neuromuscular activation under a controlled stroke-rate condition using surface electromyography (sEMG). The present findings demonstrate that resistance modulation does not uniformly influence all muscles during water-resistance rowing exercise. Instead, resistance-dependent neuromuscular adaptations appear to be muscle specific, with the biceps brachii exhibiting the greatest responsiveness to increasing resistance demands. The present findings suggest that resistance-dependent neuromuscular adaptations during water-resistance rowing were muscle specific. The biceps brachii demonstrated the greatest sensitivity to increasing resistance demands, whereas the remaining muscles, including the latissimus dorsi, exhibited relatively stable activation patterns across conditions. The results further demonstrated that muscle activation amplitudes under all resistance conditions generally remained below 40% MVIC, suggesting that water-resistance rowing represents a low-to-moderate intensity neuromuscular training modality that may provide sufficient motor-unit recruitment for functional adaptation while minimizing excessive mechanical loading and fatigue-related stress. The biceps brachii was the only muscle demonstrating significant resistance-dependent increases in activation. Relatively stable activation across the first three resistance settings followed by greater activation at the MAX condition was therefore interpreted as a muscle-specific response rather than a generalized pattern across all examined muscles. The present findings indicate that water-resistance rowing may provide an effective low-to-moderate intensity exercise modality for rehabilitation, older adults, and individuals requiring a gradual progression of resistance loading. Furthermore, resistance-dependent neuromuscular information obtained through sEMG measurements may facilitate individualized exercise prescription and support the development of intelligent exercise-monitoring systems.

The present findings further suggest that integrating sEMG with biomechanical sensing technologies may facilitate the development of intelligent exercise-monitoring systems capable of providing individualized exercise prescription and real-time rehabilitation guidance during water-resistance rowing exercise. The integration of fixed stroke-rate control with sEMG analysis provided a more objective evaluation of rowing-related neuromuscular demand by minimizing movement-speed variability. Therefore, the proposed experimental framework may serve as a practical method for evaluating exercise intensity, muscle recruitment behavior, and resistance-training characteristics in rowing-based exercise systems. Practically, the present findings may assist coaches, clinicians, rehabilitation specialists, and exercise equipment developers in optimizing resistance-control strategies and individualized exercise prescriptions. The present findings indicate that water-resistance rowing exercise produced relatively low-to-moderate neuromuscular demands in healthy young adults. These findings provide preliminary physiological information that may inform future exercise prescription strategies and suggest potential applications that warrant further investigation. Future studies should further investigate muscle coordination timing, fatigue-related adaptations, co-contraction behavior, and long-duration rowing exercise responses under different resistance and stroke-rate conditions. Additionally, integrating sEMG with inertial measurement units (IMUs), motion capture systems, and biomechanical modeling may further improve the understanding of resistance-dependent neuromuscular control mechanisms during rowing exercise. Because the present investigation exclusively recruited healthy university students with previous rowing experience, no direct conclusions can be drawn regarding older adults or rehabilitation populations. Future studies should directly evaluate the safety, feasibility, and efficacy of water-resistance rowing exercise in these clinical populations. Although the present findings suggest that water-resistance rowing may provide relatively low-to-moderate neuromuscular loading, direct recommendations for rehabilitation populations cannot be made based on the current data. Future investigations involving older adults and clinical populations are necessary before rehabilitation-specific exercise prescriptions can be established.

## Figures and Tables

**Table 1:** Two-way repeated-measures ANOVA summary for resistance level and muscle group effects on muscle activation.

Source	SS	df	MS	F-Value	*p*-Value	ηp^2^
Resistance Level	1313.70	3	437.90	8.31	<0.001 *	0.430
Error (Resistance)	1739.10	33	52.70	–	–	–
Muscle Group	16,863.80	5	3372.76	8.95	<0.001 *	0.449
Error (Muscle)	20,726.20	55	376.84	–	–	–
Resistance × Muscle	790.13	15	52.68	2.70	0.001	0.197
Error (Resistance × Muscle)	3219.15	165	19.51	–	–	–
Total	57,851.14	287	–	–	–	–

Note: * *p* < 0.05, SS = sum of squares; MS = mean square; Partial η^2^ = partial eta squared.

**Table 2:** Muscle activation under different resistance conditions (%MVIC, n = 12).

Muscle	MIN	Level 2	Level 3	MAX
Biceps brachii	24.34 ± 13.35	30.61 ± 17.92 a	34.81 ± 23.38 ab	39.37 ± 22.00 abc
Triceps brachii	8.23 ± 7.36	11.58 ± 9.35	12.73 ± 11.79	12.50 ± 11.52
Deltoid	9.72 ± 6.55	10.99 ± 5.74	13.09 ± 8.37	14.20 ± 7.59
Latissimus dorsi	20.29 ± 12.49	23.05 ± 12.00	22.44 ± 13.57	24.93 ± 13.62
Rectus femoris	10.46 ± 11.51	10.71 ± 10.96	10.98 ± 10.31	13.50 ± 10.94
Tibialis anterior	15.84 ± 7.93	15.68 ± 6.02	16.59 ± 8.27	19.74 ± 8.28

Values are presented as mean ± SD (%MVIC). a: significantly different from Level 1. b: significantly different from Level 2. c: significantly different from Level 3. Lowercase letters indicate significant pairwise differences among resistance conditions for the biceps brachii only (*p* < 0.05). No significant pairwise differences were observed for the remaining muscles.

## Data Availability

The datasets used and analyzed during the current study are available from the corresponding author on reasonable request.

## Abbreviations

The following abbreviations are used in this manuscript:

ANOVA	Analysis of Variance
BB	Biceps Brachii
DEL	Deltoid
EMG	Electromyography
Hz	Hertz
LD	Latissimus Dorsi
MVIC	Maximal Voluntary Isometric Contraction
RF	Rectus Femoris
RMS	Root Mean Square
RMS-EMG	Root Mean Square Electromyography
SD	Standard Deviation
SEMG	Surface Electromyography
SPSS	Statistical Package for the Social Sciences
TA	Tibialis Anterior
%MVIC	Percentage of Maximal Voluntary Isometric Contraction
